# Evaluating sexual violence prevention and response strategies in higher education: insights from a systematic review of North American universities

**DOI:** 10.3389/fpubh.2026.1919906

**Published:** 2026-10-21

**Authors:** Ana Vidu

**Affiliations:** Department of Sociology, University of California, Berkeley, CA, United States

**Keywords:** campus violence, effective policies, higher education, prevention actions, sexual harassment

## Abstract

**Introduction:**

The scientific literature highlights the critical need for strong university policies and protocols to prevent and respond to sexual violence. However, key gaps persist in identifying the most effective strategies, particularly for survivors, and determining which institutions lead in best practices.

**Methods:**

This systematic review examined and compared prevention and response mechanisms across 20 North American universities (comparing top-ranked and lower- ranked institutions based on ARWU 2022). The analysis evaluated factors such as the diversity of resources, formal versus informal support structures, and mandatory reporting policies.

**Results:**

The systematic review identified three key findings: (1) Universities without mandatory reporting policies prioritize community-focused actions (such as peer support and bystander training) over bureaucratic compliance. (2) Peer-led initiatives (including CARE centers and 24/7 hotlines) are the most impactful resources for survivors. (3) No correlation exists between institutional academic ranking (ARWU 2022) and policy effectiveness, directly challenging assumptions regarding elite universities’ leadership in this domain.

**Discussion:**

The study highlights key best practices (such as survivor-centered design and off-campus partnerships) while critiquing outdated measures like mandatory reporting without confidentiality safeguards. Based on these insights, it offers actionable recommendations and guidance for universities worldwide seeking to strengthen campus prevention and response efforts against sexual violence.

## Introduction

Sexual violence in academia is a pervasive and deeply concerning issue that demands urgent attention and action (1). With growing awareness of the prevalence and impact of sexual harassment within university settings, there is a pressing need for evidence-based policies and support structures to create safer and more inclusive environments (2). Drawing on existing literature and empirical data (3), this article explores the multifaceted nature of sexual violence prevention and response, highlighting the importance of peer support networks, confidential resources, and continuous evaluation of policies (4). By synthesizing findings from diverse institutions and contexts, this study offers guidance for universities worldwide seeking to strengthen their commitment to addressing sexual violence and fostering a culture of respect, trust, and support within their academic communities.

The systematic review presented in this article aims to address a critical gap in research by examining effective policies and strategies for preventing and responding to sexual violence in higher education. Despite the recognition of the need for university protocols to address sexual violence, there remains ambiguity regarding the most effective strategies, particularly for survivors, and variations in practices among different institutions. This article conducts a comprehensive analysis of prevention and response mechanisms implemented by 20 North American universities, considering factors such as the diversity of resources, formal and informal mechanisms, and the existence of mandatory reporting policies. By examining both top-ranked and lower-ranked universities, this study provides valuable insights into the strengths and weaknesses of existing approaches, offering recommendations for enhancing prevention and response efforts globally.

### Study organization

This article seeks to identify effective policies addressing sexual harassment at universities, analyze their contextual factors and characteristics, and explore methods for transferring successful policies to other institutions. A comprehensive review of guidelines from 20 US universities was conducted, with 10 employing mandatory reporting policies and 10 based on support mechanisms. Additionally, universities were selected across various ranking positions to examine potential correlations between institutional ranking and support policies. While the primary focus is on student support, attention is also given to policies for staff and academics. Through a systematic review and integration with existing literature on sexual harassment and support mechanisms in the academic context, this article aims to encourage universities to adopt effective policies, aligning with Goal 5 of the United Nations Sustainable Development Goals (5).

### Definitions and conceptual framing

In this article, I follow the United Nations definition to describe *sexual harassment* in the following terms: “Unwelcome sexual advances, requests for sexual favors, and other verbal or physical conduct of a sexual nature when (1) Submission to such conduct is made either explicitly or implicitly a term or condition of an individual’s employment, or (2) Submission to or rejection of such conduct by an individual is used as a basis for employment decisions affecting such individual, or (3) Such conduct has the purpose or effect of unreasonably interfering with an individual’s work performance or creating an intimidating, hostile, or offensive working environment.”(n.d.).

Along the same line, the World Health Organization defines sexual violence (6) as “any sexual act, attempt to obtain a sexual act or other act directed against a person’s sexuality, using coercion, by any person regardless of their relationship to the victim, in any setting. It includes rape, defined as the physically forced or otherwise coerced penetration of the vulva or anus with a penis, other body part or object.” According to RAINN (7), “sexual assault refers to sexual contact or behavior that occurs without explicit consent of the victim. Some forms of sexual assault include: (1) Attempted rape; (2) Fondling or unwanted sexual touching; (3) Forcing a victim to perform sexual acts, such as oral sex or penetrating the perpetrator’s body; (4) Penetration of the victim’s body, also known as rape.”

These definitions must be considered because different types of harassment can occur in other contexts where people develop. In the academic environment, harassment can happen between peers, meaning harassment received by a student from another student and harassment received by staff, who have some legal protections as employees (8).

In this study, “policy effectiveness” is defined in terms of the presence and configuration of survivor-centered structural features associated in the literature with improved access, safety and support. Specifically, effectiveness was operationalized through the seven predefined analytical categories (included in the *data analysis* section) derived from prior research and best-practice guidance. Within this framework, policies were considered “more effective” when they combined (a) confidential, peer-led or student-informed support, (b) alternatives to universal mandatory reporting, and (c) accessible, clearly advertised resources. These criteria align with evidence that survivor-centered, confidential and community-based mechanisms are more likely to be used and trusted by victims than solely compliance-oriented models.

### Sexual harassment in academia

Sexual harassment in academia is a deeply concerning and pervasive issue that affects students and faculty across diverse academic landscapes (9). Overcoming sexual harassment is one of the primary objectives of most universities (10–12). Much research has already demonstrated that many situations defined as sexual harassment have been happening in the academic context for several decades (13). Underreporting such cases has been among the most common acts in the academic field, making silence one of the most common realities on campuses (14). Research has also shown that many of these harassments occurred because some of the department’s faculty, staff, or people did not dare to report what they knew was happening (15). There is also a lack of support on the institutional side. The negative consequences that survivors and their supporters usually experience when complaining about a case contribute to maintaining the open secret or the deliverable indifference toward this topic (16, 17).

Numerous studies and reports underscore the widespread nature of this problem, revealing that individuals of all genders and backgrounds can be subjected to unwelcome advances, discrimination, and other forms of harassment within the university setting (18). Universities increasingly recognize the urgent need to address this systemic problem by implementing comprehensive policies, awareness campaigns, and support structures to create safer and more inclusive environments (4). Many policies and actions have been employed in universities to prevent and act against sexual harassment (12). Since the first complaints were made in the United States (19–21), anti-harassment policies began to be implemented in North American universities (22).

Years later, some of these policies were evaluated, as in the case of the bystander intervention program (23). Many studies have already demonstrated the importance of the bystander in denouncing harassment and supporting the victim (24). The situations of sexual violence happening in institutions are opportunities for bystanders to intervene. When strong foundations are being dismantled, it is because a significant movement of people is sitting behind them. As many universities make efforts to prevent and intervene in sexual violence, the peers, mates and friends of those targeted by such attacks are becoming “allies” and creating opportunities for support, assistance and solidarity (11).

Aligned with the progress to achieve gender equality in the labor market, academia cannot ignore this transformation. Some state policies advocate for equal opportunities in higher education (25). As efforts to combat sexual harassment continue, institutions globally must collaborate, share best practices, and foster cultures of respect and accountability to eradicate this pervasive challenge from academic spaces (26).

### Existing regulations

Some of the universities’ policies have been regulated by legislation such as Title IX. The Title IX Education Amendments of 1972 is a federal civil rights law that prohibits discrimination based on sex in any education program at a public institution, which is any institution that receives federal funding. Under this law, sexual discrimination is described as sexual harassment, sexual battery, sexual assault and rape. Indeed, Title IX is one of the most frequently invoked laws for complaints within the context of US universities (27). Violation of Title IX should be the complaint identified by claimants, describing the situations in which they felt that their administrators had violated what Title IX was established to defend. In cases in which harassment is proven, the university is held responsible if the institution had authority over the harasser and the environment in which the situation took place (28).

Title IX requires universities to have data on sexual harassment and discrimination (12). Title IX also requires the existence of a “responsible employee.” Many universities have implemented the mandatory reporting mechanism justifying that they are responding to Title IX, even when this legislation never required its practice (17). This is important because this reality would have to show some policy flexibility. Still, this has not been the case, even considering there is little evidence to show the efficacy of this policy (4). The mandatory reporting mechanism has long been considered necessary for cases to come to light. It has taken a lot of research, evidence, and activism for these policies to be changed (29).

Globally, universities are adopting policies inspired by U. S. models while adapting them to local legal and cultural contexts (30). Key examples include the Istanbul Convention (2011) in Europe, which obligates signatory countries to address gender-based violence in education, leading to policies like Spain’s *Organic Law 3/2007* (31), which explicitly prohibits sexual harassment in academia. The UK Equality Act (2010) mandates institutional accountability for harassment, with universities like Oxford and Cambridge establishing independent review boards (18). Another example is Australia, where the *Respect@Work Report (2020)* inspired reforms requiring universities to adopt “positive duty” frameworks, shifting the burden of prevention onto institutions (1).

### Consequences for survivors

Sexual harassment has dire consequences for those who experience it. The academic context aggravates some of these consequences (32). Beyond the physical consequences and the effects on the mental health of the victims and their supporters, in the academic context, it adds other consequences such as abandonment of the academic career, insecurity as a person, distrust as a student or scientist, disappointment with the institution itself, and even with peers (33). Some victims have even stated that it is worse to report harassment at university than to suffer harassment at university (34). Another peculiarity of the academic environment is that the perpetrator is often someone the victim knows, which makes the academy a hostile context to denounce and move on (35). The existence of these consequences, especially when they are not taken into account when analyzing complaints in the speaking up process, might lead to a silencing effect on survivors, who will tend not to speak up if they feel a lack of support (29). Some studies have shown that the lack of analysis of this reality from the point of view of the consequences for victims of speaking up increases the silence surrounding sexual harassment on campus (36).

However, despite developing multiple programs related to this problem, the percentage of sexual assault and rape continues to be high on college campuses. For this reason, more studies using different approaches are analyzing this topic to highlight the severe consequences for both victims and society, emphasizing the need for effective solutions (37, 38). Among extant literature on gender violence at universities, some highlight the role of student mobilization and informal mechanisms for raising awareness when programs that can prevent gender violence from happening are assessed (39).

### Role of networks of support

Within the mechanisms of prevention and action against sexual harassment at the university, support, as well as solidarity with the victims, programs such as bystander intervention have proven to be the most efficient way to respond to harassment situations. In this line, support for the victim is crucial in accompanying their process (10). Research has shown that support networks play an essential role in several aspects, such as breaking the silence, accompanying the victim, and pressuring universities to improve their policies (11). Support networks began to form in different universities as the only informal and confidential mechanism available to a victim suffering a situation of harassment. These support networks have become increasingly prominent, and their role has become more and more formal. Universities are increasingly recognizing the importance of fostering such networks to promote a culture of respect and inclusivity. No one now doubts the need for informal support networks to provide the support that victims need. Moreover, this support has become more formalized in some universities, as in the case of CARE centers (40).

Support networks play a crucial role in academia concerning sexual harassment. These networks usually consist of survivors, activists, stakeholders, faculty members, administrators, student organizations, as well as advocacy groups, all dedicated to creating safer environments for students and scholars (11). They provide survivors of sexual harassment with crucial avenues to report incidents, seek confidential guidance, and access resources like counseling, support groups, as well as legal assistance. These networks also engage in proactive education and awareness campaigns, helping to prevent harassment and empowering individuals to speak out against it (29). By combining education, advocacy, and supportive measures, the peer support networks work collectively to ensure that academic institutions become spaces where everyone can pursue knowledge and careers without fear of harassment (41). It is also essential to consider the support needed by the people who support the victims, which in the scientific literature is referred to as second order of sexual harassment (22). Without support for the victims, they cannot move forward, and the consequences for the supporters limit the protection of the direct victims. This is why it is also essential to protect the protectors, which is already legislated in some regions (31).

## Methods

In this study, the systematic review aims to gather data on the policies and programs that support survivors in academia, offering the most implemented actions to unveil the open secret regarding sexual harassment in academia, contributing to safer and harassment-free university campuses.

### Research questions

Despite increasing institutional commitments to addressing sexual violence, universities continue to implement highly varied, and often questioned, policies and practices. Yet, there remains a lack of clarity about which specific actions and configurations most effectively support survivors and foster safe academic environments. The research questions in this study are designed to bridge this gap by systematically examining what policies are in place, how they are shaped by contextual factors such as institutional ranking or reporting mandates, and what survivor-centered mechanisms emerge as best practices. With growing international interest in replicating effective U. S.-based strategies and critiques surrounding ineffective or harmful models (e.g., mandatory reporting without confidentiality), there is an urgent need for empirical insights into what works, for whom, and in what context. These questions not only aim to document institutional efforts but also to consider their transferability and relevance beyond the specific universities studied, offering evidence-based guidance for global higher education communities seeking to create more just and supportive environments.

Four primary research questions guided this study:

1. What actions have been implemented to support survivors in different universities across the United States?
2. What unique situational or contextual factors characterize each university’s approach to survivor support?
3. What are the most implemented support measures within each university?
4. How can the most effective support actions be disseminated to universities beyond the United States?

### Data selection

The universities chosen for the analysis were selected based on two distinct processes. First, universities were chosen based on their ranking in the 2022 Academic Ranking of World Universities (ARWU), aiming to capture a spectrum of institutions ranging from top-tier to lower-ranked establishments. Second, additional universities were selected based on their unique approaches to supporting survivors of sexual harassment, regardless of their ranking. Notably, the presence or absence of a mandatory reporting policy emerged as a distinguishing criterion. Some universities that do not have a universal mandatory reporting policy were chosen to see how mandatory reporting has been replaced by alternative support mechanisms.

Being more specific about the methodological choices, and also to mitigate potential bias from the inclusion of historically prominent universities, our sample was balanced across: (1) institutional ranking (ARWU 2022); to examine whether institutional prestige correlated with policy robustness, I included both top-tier and lower-ranked universities (e.g., Harvard [1st] to University of Nebraska [201–300]); (2) policy approach; half the institutions employing mandatory reporting policies and half using alternative support models (e.g., confidential advocacy, peer networks); (3) institutional capacity; including lower-budget universities with more limited staffing and technological infrastructure; (4) historical significance: Institutions like Yale and UC Berkeley were included due to their pioneering roles in Title IX litigation and survivor advocacy (20, 28). Subsequent analyses showed no correlation between ARWU rank and the scale or type of survivor-support initiatives, indicating that policy quality was not driven by institutional prestige alone.

Additionally, some universities were chosen because they have been home to essential cases and pioneering reports, such as Cornell University (42), the University of Yale (20), and Berkeley (28). It is also important to note that the three universities chosen for this analysis, UC Berkeley, Harvard, and Stanford, were selected because they correspond to the case studies of the research project on which this article is based, UniswithHeart.

### Data collection and search procedure

To answer the four guiding research questions listed below, a structured data collection process was designed to ensure that each question could be directly addressed through the analysis of relevant university practices, policies, and institutional contexts. Data were collected from the official websites of 20 selected North American universities listed in Table 1. For each institution, I searched for publicly available documents, policies and programs related to sexual harassment and sexual violence, including Title IX reports, diversity and inclusion initiatives, peer support service pages, and prevention training descriptions. I included documents that specified concrete policies, support mechanisms, or procedures for students and/or staff, and excluded general statements of principle without operational details. All identified materials were organized into a comprehensive spreadsheet that included variables relevant to each research question. This process resulted in a final sample of 20 universities and their corresponding policy documents. Table 1 synthesizes this dataset, linking university practices with institutional profiles.

**Table 1:** Universities, offices, and policies analyzed.

University’s name	Position in Ranking ARWU 2022	Offices in place consulted at each university (selection)	Policies in place consulted at each university (selection)	Actions in place consulted at each university (selection)	Mandatory reporting policy or not
Harvard University	1	Office of Sexual Assault Prevention and Response Title IX Office Ombuds Office	Student Handbook Mandatory Online Course Equity, Diversity and Inclusion Policy	Diversity Peer Educators 24/7 hotline Off-campus resources Harvard College Women’s Center	Yes
Stanford University	2	Ombuds Office SHARE Title IX Office Office of Diversity, Inclusion, and Belonging	Policies, Procedures and Resources Different training for Personnel, Students, and Graduates	Sexual Violence Supportive Measures Confidential Support Team (CST)	Yes
Massachusetts Institute of Technology (MIT)	3	Institute Discrimination and Harassment Response Office (IDHR) Title IX Office Ombuds Office	Policy on Harassment Graduate Handbook Title IX Sexual Harassment Policy	Confidential Resources (Health Care, Clergy, Counseling) Peer Group Resources CARE Team	Yes
University of California, Berkeley	5	Office for the Prevention of Harassment and Discrim Title IX Office Ombuds Office (Faculty/Staff and Students/Postdocs)	Sexual Violence and Harassment Prevention Training	PATH to Care Women’s Center Gender Equity Resource Center	Yes
Princeton University	6	University Sexual Misconduct/Title IX Coordinator SHARE Office Ombuds Office	Sexual Harassment/Assault Policies Preventing Sexual Harassment Program	Confidential Resources (medical, religious, counseling) Supportive Measures for Complainants /Respondents Carebridge (Assistance Program)	Yes
Columbia University	8	Title IX Office Ombuds Office Center for Student Success and Intervention	University Policy for Gender-Based Misconduct Interim Title IX Policies	Confidential Resources (counseling, health care) Training Materials	Yes
California Institute of Technology	9	Equity and Title IX Office Staff and Faculty Consultation Center Ombuds Office	Sex- and Gender-Based Misconduct Policy Unlawful Harassment and Abusive Conduct Policy	Campus Sexual Violence Advocate Student Wellness Counseling Services	No
Yale University	11	SHARE Center Title IX Coordinators Deans Designees for Harassment/DEI	Required Annual Training University-Wide Committee on Sexual Misconduct	LiveSafe App (Anonymous Reporting) 24/7 Community Resource	Yes
Cornell University	12	Office of Institutional Equity and Title IX Women’s Resource Center LGBT Resource Center Ombuds Office	Prohibited Bias, Discrimination, Harassment Policy	24/7 Consultation Cornell Victim Advocacy Program Faculty and Staff Assistance Program (FSAP)	Yes
University of Washington	18	Title IX Coordinator Office Ombuds Office	Policy on Sex/Gender-Based Violence Student Conduct Policy	Mutual No Communication Directives (MNCDs) Training Upon Request LiveWell Confidential Advocates	No
University of Michigan- Ann Arbor	26	Equity, Civil Rights, and Title IX Office (ECRT) Ombuds Office	Sexual/Gender-Based Misconduct Policy Violence Prevention Programs	Bystander Training for Incoming Students Survivor Support	No
New York University	28	Title IX Coordinator Office of Equal Opportunity Office of Community Standards	Sexual Misconduct, Relationship Violence, and Stalking Policy	Wellness Exchange Hotline and Chat App Crisis Response Counselor (CRC)	No
University of North Carolina Chapel Hill	31	Title IX Office Ombuds Office Gender Violence Services Coordinators (GVSCs)	Policy on Prohibited Discrimination/Harassment	Counseling and Psychological Services Orange County Rape Crisis Center (OCRCC)	No
Brown University	98	SHARE for Students Ombuds Office DEI Office	Sexual/Gender-Based Harassment and Violence Policy	Peer Support Off-Campus Hotline	No
The George Washington University	201–300	Title IX Coordinator Equal Employment Opportunity Office Ombuds Office	Sexual Assault and Intimate Violence (SAIV) Policy	Mandatory Training	No
University of Nebraska	201–300	Title IX Office Ombuds Office CARE Centers	Sexual Misconduct Policy	24/7 Hotline Mandatory Training Courses	No
University of Oregon	401–500	Title IX Office Ombuds Office Women’s Center	Workplace Harassment and Discrimination Prevention Policy	Mandatory Training Peer Support	No
CUNY Graduate School	401–500	Title IX Office Ombuds Office DEI Office	CUNY Policy on Sexual Misconduct	Training RAINN Hotline	No
University of New Mexico	501–600	Title IX Coordinator Office of Equal Opportunity (OEO) Ombuds Office	Sexual Harassment/Assault Interim Policy	Rape Crisis Center (Confidential) LoboRESPECT Advocacy Center	Yes
Tulane University	501–600	Title IX Office Office of Institutional Equity (OIC)	Sexual Misconduct Resource Guide The Well for Health Promotion—Campus Health	Sexual Aggression Peer Hotline and Education Training Courses	Yes

Own elaboration.

*RQ1*: (What actions have been implemented to support survivors?) was addressed by cataloging all existing programs, services, and policies related to survivor support (e.g., peer support groups, 24/7 hotlines, confidential advocacy services).

*RQ2*: (What unique situational or contextual factors characterize each university’s approach?) was explored by gathering data on institutional characteristics such as ranking (ARWU 2022), resource allocation (budgets), staffing levels, and technological infrastructure for prevention and response systems.

*RQ3*: (What are the most implemented support measures within each university?) was addressed through a comparative framework of recurring support mechanisms across all universities, enabling identification of the most common elements (e.g., mandatory training, ombuds offices, peer-led programs).

*RQ4*: (How can the most effective actions be disseminated to other universities?) was informed by identifying exemplary cases of formalization of support structures (e.g., CARE centers), scalable models (e.g., partnerships with off-campus crisis services), and alternatives to mandatory reporting policies.

### Data analysis

To systematically answer each research question, a thematic content analysis was conducted based on a framework that aligned directly with the study’s aims. The data were analyzed across seven predefined categories, chosen to address the study’s central concerns. The seven categories are the following:

1. Distinctions between policies for students and staff.
2. Presence or absence of mandatory reporting policies.
3. Formal incorporation of informal support mechanisms.
4. Provision of mandatory harassment training.
5. Accessibility of support through external organizations.
6. Availability of confidential resources and peer support networks.
7. The existence of 24/7 consulting hotlines.

For RQ1, I reviewed each university’s available policies and services to extract a full list of survivor-support actions. These were thematically grouped (e.g., “confidential advocacy,” “bystander training,” “CARE centers”) and coded for presence or absence.

For RQ2, I- conducted a comparative institutional analysis, assessing how ranking, funding levels, staffing, and technological systems (e.g., anonymous reporting platforms) influenced the nature and depth of survivor support.

For RQ3, I quantified the frequency and distribution of support measures across the sample to identify the most implemented strategies. This included tracking the number of universities implementing each type of service or policy.

For RQ4, I conducted a best-practice extraction by identifying policies and models that had been successfully institutionalized or demonstrated replicability potential (e.g., survivor-led policy reviews, off-campus collaborations). These cases were cross-analyzed for their alignment with existing literature and their adaptability across institutional contexts.

In this way, the analytical process ensured that each research question was explicitly and systematically addressed, allowing for both descriptive and comparative insights.

### Scope and limitations of the research

Specific research has been conducted for each of the 20 universities selected, considering factors such as (1) the budget allocated to harassment issues, DEI, or mechanisms for supporting survivors; (2) their technological infrastructure; (3) the number of employees and their competencies. Drawing on this, a comparative capacity assessment has been conducted.

Regarding budget allocation, the results of this search show that five universities (Harvard, Stanford, University of Michigan, UC Berkeley and Cornell University) are considered higher-capacity institutions (Top 5). They allocated between 10 million and 5 million dollars for Title IX offices, DEI offices, bystander training, and survivor support measures. On the other side, there are five lower-capacity institutions (bottom 5) (University of Nebraska, CUNY Graduate School, G. Washington University, University of Oregon and Brown University) that allocate between $1 M and $300 K for Title IX offices, DEI initiatives, prevention programs and confidential advocates.

Considering the technological infrastructure, top-ranked universities tend to invest in integrated tech solutions, while others lag due to cost barriers. I distinguish: (A) advanced infrastructure Yale, Stanford, Harvard: use AI-driven platforms (e.g., *LiveSafe app*) for anonymous reporting, real-time threat assessment, and training modules. Columbia, Cornell: integrated case-management systems (e.g., *Maxient*) to track complaints and ensure compliance. (B) Limited infrastructure University of Washington, Tulane: basic online reporting forms; no centralized tracking. CUNY, University of Nebraska: outdated systems; paper-based processes still common.

Concerning the number of employees and their competencies, the research shows: (A) specialized staffing Harvard, MIT: Employ 15–20 full-time Title IX professionals, including trauma-trained investigators and psychologists. UC Berkeley, Princeton: Dedicated “Prevention Specialists” with public health backgrounds. (B) generalized staffing University of New Mexico, Oregon: 1–2 Title IX coordinators handle all cases; high turnover. Brown, NYU: Rely on adjunct counselors or graduate students for survivor support. In general, institutions with dedicated, trained personnel resolve cases faster and retain more survivors (43).

This study shows some limitations. (A) Data availability, as I rely on publicly reported data (e.g., university websites, Title IX reports) may overlook unpublished initiatives or internal challenges. Budget figures and staffing details were often inconsistently disclosed. (B) Selection bias, as the inclusion of historically prominent universities (e.g., Harvard, Yale) may bias trends toward elite institutions, despite efforts to diversify rankings. (C) Methodological constraints, as no primary survivors were interviewed. The effectiveness of policies was inferred from institutional actions rather than direct survivor experiences.

## Findings

To provide a coherent and accessible account of the findings, this section is organized into two parts. The first one provides an overview following the four research questions guiding the study. Each subsection presents the key results related to one research question, using the predefined analytical categories to frame the data. These categories were developed from the literature and applied consistently across all cases, allowing for both within- and cross-institutional analysis.

For RQ1 (actions implemented to support survivors), findings focus on institutional structures such as Title IX, DEI, and SHARE offices; the development of peer support and CARE centers; the provision of harassment training; and the availability of confidential resources. Some of the findings in this regard are the following:

1. The context of anti-harassment offices (Title IX offices, Ombuds, DEI, SHARE). These institutional actions represent the core structures universities use to support survivors.
2. Informal support converted into formal support (CARE centers, peer support networks). It shows evolution from grassroots initiatives into institutionalized survivor support systems.
3. Provision of mandatory harassment training (Bystander training, peer-led education). It represents proactive educational measures implemented for community prevention and awareness.
4. Availability of confidential resources and peer support. It demonstrates accessible, survivor-centered services across institutions.

For RQ2 (contextual factors shaping responses), themes include the extent to which institutions update policies based on survivor feedback and evidence; the influence of staffing and technological infrastructure; and the (non-)correlation between university ranking and support quality. Some of the findings in this regard are the following:

1. Lack of policy updates based on evidence (second order of sexual harassment). It reveals disparities in how universities respond to recent literature and survivors’ voices.
2. Concerning technological and staffing infrastructure (differences in reporting platforms, staff training levels). It highlights how institutional capacity shapes the scope and quality of support mechanisms.
3. The relationship between being a top university and the support offered to the victims. It examines whether prestige correlates with policy quality and responsiveness (finding: it does not).

For RQ3 (most implemented support measures), I examine how institutions balance mandatory reporting policies with support-based alternatives, and how prevention and peer-led education are integrated into campus life. Some of the findings in this regard are the following:

1. Mandatory reporting vs. support policy. It assesses the most frequently applied strategies and contrasts their impacts (mandatory vs. support-based).
2. Configuration of mandatory support. It shows how institutions without a mandatory reporting structure for survivor support (e.g., student-informed policies).
3. Training and prevention education. It identifies widely adopted tools for raising awareness and empowering campus communities.

For RQ4 (dissemination of effective practices), findings highlight scalable and transferable initiatives, such as external partnerships (e.g., with RAINN or local rape crisis centers), the formalization of student-led programs, and inclusive, survivor-centered policies. Some of the findings in this regard are the following:

1. Connecting universities to off-campus resources (RAINN, local rape crisis centers, external counseling partnerships). It demonstrates scalable, adaptable models for universities with fewer internal resources.
2. Formalization of peer-led programs (e.g., CARE Centers). It represents a transferable best practice born from student activism and institutional adaptation.

Use of inclusive, community-centered practices. They consist of merging models that prioritize survivor agency over bureaucratic mandates.

On the other hand, the systematic review results revealed several critical insights into the landscape of survivor support mechanisms within North American universities. These findings are elaborated upon in the subsequent sections, aligning with the identified analytical categories and supplemented by relevant literature discussions.

### The context of anti-harassment offices

According to our analysis, all the universities have a Title IX Office and a Title IX coordinator, thus complying with the law on universities in the United States. The 20 universities analyzed also have an Ombuds Office. In line with the policies implemented more recently in universities, such as the Diversity, Equity, and Inclusion offices, almost all (except three) of the universities in this study have a DEI office. The SHARE office, with whatever name it has in each university (e.g., “Office of Sexual Assault Prevention and Response”), is another reality in almost all the universities. Only seven of the universities analyzed do not have an office against harassment but include this issue under other offices such as *“Institutional Equity or Equal Opportunity.”*

Regarding training, most of the universities studied (14) have mandatory training courses. Many of these trainings offer tools for being an active bystander and different support mechanisms. These resources include materials and training on acting if a case of gender-based violence is present or known to occur. Top-ranked universities combine mandatory training with specialized hotlines, while others (e.g., CUNY) rely on general counseling services. For instance, Harvard University has “diversity peer educators,” organizing student-led workshops on consent and bystander intervention, and a 24/7 confidential hotline, staffed by trauma-trained professionals. Stanford University has the SHARE Office which offers a “Confidential Support Team” (CST) for immediate crisis response and “active bystander training,” which is mandatory for all incoming students, using scenario-based modules. The University of Michigan has the SAPAC (Sexual Assault Prevention and Awareness Center) offering peer-led support groups like *Healing Spaces* for marginalized communities.

### Lack of policy updates based on evidence

The research described in this manuscript also focused on analyzing the content of existing policies and updating them based on the latest scientific literature on sexual harassment in the academy. While most programs and procedures for addressing complaints and harassment are detailed, certain aspects are still missing in many policies. One key aspect is the issue of second order of sexual harassment (44), which refers to the negative consequences (retaliation, attacks, etc.) that may be suffered by those who support victims. Research has shown that fear is the leading cause that limits most people from supporting victims (15). Therefore, since victims need support, it is crucial to implement protection policies and measures to support those who support them.

Only 4 of the 20 universities analyzed (e.g., Oregon, Michigan, UC Berkeley, Cornell) formally integrate survivor feedback into policy updates. At the University of Oregon, there is a *Survivor-led policy review*, which consists of annual forums to update protocols (e.g., added second order of sexual harassment protections in 2022). At UC Berkeley, the PATH to Care Center has adopted trauma-informed practices only after student protests (2021). At Cornell University: Delayed Reforms retained mandatory reporting until 2023 despite student demands for confidentiality.

### Informal support converted into formal support

Informal support is one of the primary mechanisms on which this study focuses. Peer support has proven vital in supporting victims and bringing about institutional change. Informal support, such as student support networks, has long been the key to fighting campus bullying (11). In other words, the place where more often victims feel listened to, believed, and supported is a space of trust and confidentiality that has made a difference for many victims in their academic careers. In this sense, an interesting aspect is that the CARE Centers were born as informal support due to student mobilizations and became formal support in several universities today. These Centers offer different support measures, such as support groups, a 24/7 hotline, and confidential advocates.

Only two of the universities studied have CARE centers as such. Still, almost all of them have peer support offices, diversity peer educators, and different peer support mechanisms, such as the Women Centers, present in 9 of the universities analyzed. Peer networks (e.g., Tulane’s hotline) show higher survivor engagement than bureaucratic Title IX offices. For instance, at Tulane they have a *Sexual Aggression Peer Hotline*: 100% student-operated, with university-funded training. At the University of Washington, there is the LiveWell Advocates program, which consists of paid positions for survivors-turned-advocates.

### Mandatory reporting vs. support policy

For many years, even decades, the mandatory report has been present in almost all universities in the United States. During the last few years, this mechanism has been questioned from the victims’ point of view and the support they receive or do not receive; as well as from the point of view of the consent of their information (45). Half of the universities analyzed in this article have implemented the mandatory report policy, and the other half do not. I examined the difference between these two situations. After analyzing the two types of universities, I conclude that the universities that do not have mandatory reporting are based more on “mandatory” support policies. That is, they do more activities for the community - non-mandatory universities average 3x more prevention activities (e.g., workshops, film forums)-, sometimes allocating fewer resources and having fewer Centers and offices, but they carry out more concrete actions (4) and are easier to find on the web for the whole community (29). An example is the University of Michigan, the University of New Mexico, Tulane University, and Brown University which have a policy for undergraduates focused on support.

To be more specific, the University of Michigan (no mandatory reporting) implements bystander training for all incoming students; hosts survivor support groups and partners with local rape crisis centers; offers EverFi courses (such as AlcoholEdu, Sexual Assault Prevention) as mandatory prevention education. On the other side, Tulane University (no mandatory reporting) prioritizes peer-led initiatives (e.g., *Sexual Aggression Peer Hotline*); and provides informal resolution options (e.g., mediation, no-contact directives). The University of Oregon also had a policy shift (45), which shows concrete improvements in survivor outreach after dropping mandatory reporting.

### Configuration of mandatory support

For those universities that do not have mandatory reporting, the basis is support and trust. I interested to see how support is configured in these universities. Our research shows that students play a crucial role. For instance, policies are student-informed in universities where supportive policies are in place, and all activities are focused on supporting their students. For instance, Princeton University contains the SHARE Office and offers “supportive measures” (e.g., academic accommodations) even without formal complaints. Beyond this and regarding prevention, they offer a series of activities, open spaces for dialogue, and film forums to talk about the issues that most concern students, such as consent, nightlife, and how to have fun while being safe at the same time. One interesting fact research has shown is that universities that do not have mandatory reporting as universal do not mean they have eliminated it. Some people and specific positions are still obliged to report any case they are aware of on campus. For example, the University of Nebraska has a Responsible Employee, a designated reporter, a confidential employee, and a student-directed employee, as is the case of the University of Oregon, which has recently changed its policy, being the first university to eliminate the mandatory Report from its policy. Other universities, such as Cal Tech and the University of Nebraska, follow this model.

### The relationship between being a top university and the support offered to the victims

I have done a metric of ranking universities with their commitment to anti-harassment to inquire if there is a connection between being an outstanding university and having excellent policies. Our findings do not show a correlation between these two variables. This shows that there is no need to be a top university to offer great support for victims of sexual harassment at your university. Put another way, the universities that are not in the highest-ranking positions also provide good supportive measures. For instance, the University of Oregon (ranked 401–500) pioneered survivor-led policy reforms. In the same vein, contrary to initial expectations, this research has found no relationship between universities’ scientific research performance and their sexual violence/harassment response policies. One interesting fact found in this regard is that having support policies is independent of being higher or lower in the ranking, but rather of having a mandatory report or support policy. For example, the University of Washington ranks high and does not have mandatory reporting (it never had), but support policies. I have also found that having more resources is unnecessary to have more and better policies. In other words, universities with fewer resources also offer excellent sexual harassment support to their community. Nor does being a public university or a private university make a difference in terms of support. There is no correlation between the ARWU rank and the number of support initiatives.

### Connecting universities to off-campus resources

It has been exciting to note that some universities recommend among their sexual harassment prevention and response policies other resources beyond the ones they offer on campus. For example, universities without a 24/7 hotline recommend that their students use the hotlines of other national organizations, such as RAINN. There are different universities, such as UC Berkeley, Harvard, Nebraska, or George Washington University; even though they have a 24/7 line, they recommend their community uses off-campus resources, such as the “Rape Crisis Centers.” This is also interesting, as the university knows that some victims may rely more on off-campus resources. In some cases, there are people from the University or students who work in these Centers outside of the university, and in other cases, they volunteer. This creates a connection between the universities and the communities to which those universities belong beyond campuses. For instance, Harvard partners with Boston Area Rape Crisis Center for off-campus counseling, and the University of Nebraska advertises RAINN Hotline due to lack of in-house resources.

## Discussion

The discussion section offers a comprehensive synthesis of the study’s findings, contextualizing them within the broader discourse on sexual harassment prevention and response in academia. Key themes such as the role of peer support networks, the evolution of formal and informal support mechanisms, and the impact of mandatory reporting policies are explored in depth. The discussion also highlights areas for improvement and future research directions.

Over the years, many existing mechanisms in universities have come about due to legislation implemented in the universities (46). Others resulted from unprecedented student mobilizations (11, 28, 29). This article discusses these measures with the scientific literature on the subject. This systematic review employed a rigorous, multi-dimensional framework to evaluate sexual violence prevention and response strategies across 20 North American universities. The selection criteria, analytical categories, and measurement approaches were designed to capture both quantitative and qualitative dimensions of institutional policies, ensuring a comprehensive assessment of their efficacy and accessibility.

Drawing on this, this article responds to the social aim of achieving scientific environments without harassment (43) by analyzing which policies best support students in American universities. This research fundings extend prior work showing that student agency, rather than institutional rank, drives effective support (11). In our sample, student-led initiatives (e.g., Tulane’s hotline) were more accessible and frequently used than top-down, compliance-oriented offices. Whereas some studies emphasize formal Title IX structures as the primary response mechanism, our results suggest that non-mandatory universities often allocate more resources to prevention (e.g., Michigan’s SAPAC) than to compliance staff. For lower-budget institutions, our evidence supports previous recommendations that affordable, scalable tools (e.g., LiveSafe-like apps) can help bridge gaps in formal infrastructure.

After analyzing in depth the mechanisms of prevention and response to any sexual harassment situation in 20 North American universities, this study shows that the key to support is peer support, and all the diversity support programs universities have student-informed. One of the most successful aspects in this regard is the CARE centers system. They were built as informal support due to the active student movement and have become formal support since the universities consider them to be a crucial part of their response. These centers are confidential and carry out essential activities such as support groups, 24-h hotlines, confidential advocate services, as well as counseling and mental health.

One example of how protection and support measures could be improved is by introducing protection measures for the people who support the victims. Although universities include bystander intervention, they do not consider the need to protect the protectors. Another element that should be implemented in more universities is that of support centers based on confidential peer support, as these are the ones that victims use the most. This also helps avoid disappointment with the university system (47).

This article is helpful because it provides tools for other universities that want to improve and update their anti-harassment policies to make them more efficient. The silence has been broken in different universities across the globe (43), which look to the US for answers and effective policies to implement in their institutions (30). While this study focuses on U. S. universities operating under the Title IX framework, the findings may offer illustrative insights for higher-education institutions in other countries. Policies and support mechanisms identified here (e.g., confidential peer-led services, bystander training, partnerships with off-campus crisis organizations) could inform discussions in other contexts, provided they are adapted to local legal frameworks, cultural norms, and institutional structures.

## Conclusion

The systematic review presented in this article offers a comprehensive examination of policies and strategies for addressing sexual violence in higher education. By analyzing the responses of 20 North American universities, the study provides valuable insights into effective approaches to prevention and support. Through a combination of data collection, analysis, and integration with existing literature, the study identifies key elements of successful policies and highlights areas for improvement.

One significant finding of the study is the importance of peer support networks and confidential resources in aiding sexual violence survivors. Establishing CARE Centers or similar hubs demonstrates the value of creating safe spaces for reporting, counseling, and advocacy. By expanding and formalizing these support mechanisms, universities can better meet the needs of survivors and foster a culture of trust and support within the academic community.

Furthermore, the study underscores the need for continuous evaluation and improvement of policies and interventions. By staying informed about the latest research findings and legal developments, universities can ensure that their approaches remain relevant and effective. Collaboration and knowledge sharing among institutions are also crucial for promoting best practices and addressing the complex challenges of sexual violence. Overall, the findings of this study provide valuable recommendations and guidance for universities seeking to strengthen their prevention and response efforts, ultimately contributing to safer and more inclusive campus environments for all.

### Policy recommendations

The systematic review presented in this article sheds light on effective policies and strategies for preventing and responding to sexual violence in higher education. Based on the findings and discussion provided, the following recommendations and guidance can be offered:

1. *Definition and scope clarity*: The most effective policies are the ones that clearly define what constitutes sexual harassment and violence within the academic context. This includes understanding various forms of harassment and ensuring that the protocol covers all relevant aspects.
2. *Integration of evidence*: Keep the policies updated with the latest international scientific research on sexual harassment and violence. Incorporating evidence-based practices into policies ensures their effectiveness and relevance.
3. *Contextual review*: Regularly review and update policies to align with evolving legal frameworks and contextual factors. Consideration should be given to the unique circumstances of each institution and its community.
4. *Support mechanisms*: Identify and establish support mechanisms both on-campus and off-campus. This includes peer support networks, counseling services, helplines, and partnerships with external organizations specializing in sexual violence prevention and response.
5. *Expansion and Support Centers*: Expand the model of CARE Centers or similar support hubs that offer confidential and comprehensive support services to survivors. These centers should provide a safe space for reporting, counseling, advocacy, and resource referral.
6. *Mandatory Reporting evaluation*: Reevaluate the effectiveness and implications of mandatory reporting policies. Consider alternatives or complementary approaches that prioritize survivor support and confidentiality while still fulfilling legal obligations.
7. *Training and awareness*: Implement mandatory training programs for faculty, staff, and students on bystander intervention, consent, and recognizing and responding to sexual harassment and violence. Raise awareness through campaigns and educational initiatives to foster a culture of respect and accountability.
8. *Continuous improvement:* Regularly assess the effectiveness of policies and interventions through data collection, feedback mechanisms, and evaluation. Adapt and refine strategies based on the evolving needs and challenges within the academic community.
9. *Collaboration and knowledge sharing:* Foster collaboration and knowledge sharing among universities, both nationally and internationally. Establish platforms for sharing best practices, lessons learned, and innovative approaches to sexual violence prevention and response.
10. *Inclusivity and equity:* Ensure that policies and support services are inclusive and accessible to all academic community members, including marginalized and underrepresented groups. Address intersectional factors such as race, gender identity, sexual orientation, and disability in prevention and response efforts

Bridging gaps between policy and practice, the recommendations align with three critical shifts in the global context of campus sexual violence. (1) From compliance to survivor-centered design. The call to replace mandatory reporting with *mandatory support* (4) reflects a growing critique of Title IX’s overemphasis on institutional liability. The focus on CARE Centers and peer networks echoes successful models in Australia (*Respect@Work*, 2020) and Spain (31) where survivor agency drives policy. (2) Prevention as developed as infrastructure, as the global trend makes the push for bystander training and consent education mirrors the EU’s Istanbul Convention standards, which mandate primary prevention. However, the research emphasis on funding reallocation (e.g., from Title IX offices to student-led initiatives) challenges the status quo in under-resourced institutions. (3) Intersectionality as a policy imperative, the unaddressed gap, while the above recommendations nod to inclusivity, global frameworks explicitly require race, gender identity, and disability audits - a step further that needs to be addressed beyond the current research.

## Data Availability

The raw data supporting the conclusions of this article will be made available by the authors, without undue reservation.
